# Oestrogen‐related receptor alpha mediates chemotherapy resistance of osteosarcoma cells via regulation of ABCB1

**DOI:** 10.1111/jcmm.14123

**Published:** 2019-01-04

**Authors:** Yantao Chen, Kunshui Zhang, Yang Li, Ruilian Guo, Kelin Zhang, Guifang Zhong, Qing He

**Affiliations:** ^1^ Orthopaedics Department Sun Yat‐sen Memorial Hospital Sun Yat‐sen University Guangzhou China; ^2^ Department of Pharmacy Sun Yat‐sen Memorial Hospital Sun Yat‐sen University Guangzhou China; ^3^ Pediatric Hematology & Oncology Sun Yat‐sen Memorial Hospital, Sun Yat‐sen University Guangzhou China; ^4^ SICU Department Sun Yat‐sen Memorial Hospital Sun Yat‐sen University Guangzhou China

**Keywords:** ABCB1, ERRα, miR‐9, osteosarcoma, SP3

## Abstract

Chemotherapy resistance is one of the major challenges for the treatment of osteosarcoma (OS). The potential roles of oestrogenic signals in the chemoresistance of OS cells were investigated. As compare to the parental cells, the doxorubicin and cisplatin (CDDP) resistant OS cells had greater levels of oestrogen‐related receptors alpha (ERRα). Targeted inhibition of ERRα by its specific siRNAs or inverse agonist XCT‐790 can restore the sensitivity of OS resistant cells to chemotherapy. This might be due to that si‐ERRα can decrease the expression of P‐glycoprotein (P‐gp, encoded by ABCB1), one important ABC membrane transporter for drug efflux. XCT‐790 can decrease the transcription and mRNA stability of ABCB1, while had no effect on protein stability of P‐gp. ERRα can bind to the transcription factor of SP3 to increase the transcription of ABCB1. Furthermore, XCT‐790 treatment decreased the expression of miR‐9, which can bind to the 3′UTR of ABCB1 and trigger its decay. Collectively, we found that ERRα can regulate the chemoresistance of OS cells via regulating the transcription and mRNA stability of ABCB1. Targeted inhibition of ERRα might be a potential approach for OS therapy.

## 1 INTRODUCTION

Osteosarcoma (OS) is an aggressive and common type of solid bone tumours in children and adolescence.^1^ Surgical resection, chemotherapy and radiotherapy have been considered as the standard treatment strategies for OS.^2^ With the improvement of therapy approach, the mortality rate of OS has a huge decline in these two decades.^3^ The 5‐year survival rate of OS patients is about 70% within the past decades.^4^ However, chemotherapy resistance, defined as tumour cells can develop resistance to a wide variety of anticancer drugs, is one of the most important formidable obstacles in the OS treatment.^2^ Doxorubicin (Dox), cisplatin (CDDP), methotrexate and ifosfamide are the first‐line chemotherapy drugs for OS patients.^5^ Once the patient was resistance to these drugs, there are no established second‐line chemotherapy drugs anymore.^1^ Therefore, studies about the mechanisms responsible for chemoresistance of OS cells would be great important for developing effective therapies for OS patients.

The development of drug resistance is associated with multiple mechanisms. The most important mechanism responsible for cancer chemoresistance is the dysregulation of ABC membrane transporters.^6^, ^7^ Among all members of ABC family involved in chemoresistance, P‐glycoprotein (P‐gp), encoded by ABCB1 (also named MDR1), is highly expressed in the drug resistant cell lines to pump out the intracellular drugs.^8^ The down‐regulation of ABCB1 can sensitize OS cells to chemotherapy drugs such as Dox and CDDP.^9^, ^10^, ^11^ Although the transcription regulation of ABCB1 is far from being completely understood, numerous studies indicated that transcription factors such as c‐jun, c‐fos, NF‐kB (p65) and Sp3 can bind to its promoter to regulate the transcription.^12^ As to the protein, P‐gp is a relatively stable protein with a half‐life of 14‐17 hours, while modification by ubiquitin is important for degradation of P‐gp.^13^ Besides, ABCC1 (also named MRP1), ABCC2 (MRP2), ABCC3 and ABCG2 (BCRP) can also contribute to drug resistance of OS cells.^14^, ^15^

It has been reported that oestrogenic signals can regulate the progression of various cancers including chemotherapy resistance.^16^, ^17^, ^18^ For example, IL‐1β induced methylation of ERα is correlated with the chemoresistance in breast cancer cells.^19^ E2‐induced CDDP chemoresistance depends on the balance between ERα and ERβ expression and the p53 pathway.^18^ Oestrogen‐related receptors alpha (ERRα), which has similar structure with oestrogen receptor (ER), can bind to ERR‐response elements (ERREs) and mediate the oestrogenic response in cells.^20^ It has been reported that ERRα can confer methotrexate resistance of OS cells via attenuation of reactive oxygen species and p53 mediated apoptosis of OS cells.^21^ Furthermore, ERRα can mediate the metabolic adaptations driving lapatinib resistance in breast cancer.^22^ The reprogramming of ERRα target gene landscape can trigger the tamoxifen resistance of breast cancer cells.^23^ Inhibition of ERRα by inverse agonist XCT‐790 induces cell death in chemotherapeutic resistant cancer cells.^24^ Our previous study revealed that ERRα participates transforming growth factor‐β (TGF‐β) induced epithelial‐mesenchymal transition (EMT) of OS cells.^25^ However, the roles of ERRα in drug resistance of OS cells were not well illustrated.

In this study, we found that the expression of ERRα was increased in Dox and CDDP resistant OS cells. Targeted inhibition of ERRα can restore the chemosensitivity of OS cells via down‐regulation of ABCB1. The transcription factor SP3 and miR‐9 were involved in ERRα regulated expression of ABCB1 in OS cells.

## 2 MATERIALS AND METHODS

### 2.1 Cell culture and establishment of resistant cells

The human OS cell line MG‐63 and HOS were purchased from the Cell Bank of Type Culture Collection of Chinese Academy of Sciences (Shanghai, China) and maintained in Dulbecco's modified Eagle's medium (GIBCO‐BRL, Grand Island, NY, USA) containing 10% fetal bovine serum (FBS; GIBCO), 2 mmol/L‐glutamine, penicillin (100 U/mL), and streptomycin (100 μg/mL) in a 5% CO_2_ humidified atmosphere at 37°C. To establish Dox resistant OS cells, MG‐63 and HOS cells were treated with Dox in a stepwise manner from 10 to 500 nmol/L over a period of 6 months according to the previous study.^26^ The Dox resistant cells were named as MG‐63/Dox and HOS/Dox, respectively. The MG‐63/CDDP cells were established similarly. Cells were incubated in drug‐free medium for 3 days before experiments.

### 2.2 Drug sensitivity assay

The chemotherapy sensitivity of OS cells was analyzed by use of the Cell Counting Kit‐8 (CCK‐8) (Beyotime, Haimen, China) according to the manufacturer's instructions. Briefly, cells were seeded at a density of 4 × 10^3^ well into 96‐well plates. After treatment and incubated with different anticancer drugs at varying concentrations, 10 μL CCK‐8 was added to each well and cultured for an additional 2 hours. The absorbance at 450 nm was measured by using a microplate reader (Model‐550; Bio‐Rad Laboratories, Hercules, CA, USA). Each experiment was performed in triplicate.

### 2.3 Real time PCR for mRNA

Cells were treated with the AxyPrep™ Multisource Total RNA Miniprep kit (Axygen Biosciences, Union City, CA, USA) to extract total RNAs. The complementary DNA (cDNA) was synthesized by use of 500 ng total RNA and PrimeScript™ RT reagent kit (Takara, Shiga, Japan). The real time PCR was conducted by use of the SYBR Premix Ex Taq™ kit (TaKaRa), an ABI 7500 Sequencing Detection System, and primers: ERRα, forward 5′‐AGGGTTCCTCGGAGACAGAG‐3′; reverse; 5′‐TCACAGGATGCCACACCATAG‐3′; ABCB1, forward 5′‐GGGAGCTTAACACCCGACTTA‐3′; reverse; 5′‐GCCAAAATCACAAGGGTTAGCTT‐3′; ABCC1, forward 5′‐GTCGGGGCATATTCCTGGC‐3′; reverse; 5′‐CTGAAGACTGAACTCCCTTCCT‐3′; ABCC2, forward 5′‐CCCTGCTGTTCGATATACCAATC‐3′; reverse; 5′‐TCGAGAGAATCCAGAATAGGGAC‐3′; ABCC3, forward 5′‐CACCAACTCAGTCAAACGTGC‐3′; reverse; 5′‐GCAAGACCATGAAAGCGACTC‐3′; ABCG1, forward 5′‐GGGGTCGCTCCATCATTTG‐3′; reverse; 5′‐TTCCCCGGTACACACATTGTC‐3′; β‐actin, forward 5′‐CCAACCGCGAGAAGATGA‐3′; reverse; 5′‐CCAGAGGCGTACAGGGATAG‐3′. The relative expression of genes was normalized to β‐actin by the 2^−ΔΔCt^ method.

### 2.4 Real time PCR for miRNA

RNAs were extracted by use of Trizol according to the manufacturer's protocol and reverse transcripted to cDNA by use of the One Step PrimeScript^®^ miRNA cDNA Synthesis kit. The expression of miRNAs was determined by comparative CT method (RQ = 2^−ΔΔCT^) and normalized to U6 level. The primers were: miR‐9: ATAAAGCTAGATAACCGAAAGT; miR‐200C: TAATACTGCCGGGTAATGATGGA; miR‐206: TGGAATGTAAGGAAGTGTGTGG; miR‐495: AAACATGGTGCACTTCTT; and U6: GCAAGGATGACACGCAAATTC. The reverse primer was provided with the kit.

### 2.5 Western blot analysis

After treatment, cells were homogenized and lysed by use of cold RIPA buffer. Next, 30 μg of total protein was separated with a 4%‐15% SDS‐PAGE gel and then transferred to PVDF membrane (Thermo Fisher, Boston, MA, USA). The membrane was blocked in PBS with 5% nonfat dried milk (Yili, Beijing, China) for 1 hour before incubated with primary antibody overnight at 4°C. The antibodies against ERRα (1:1000, sc‐65718), GAPDH (1:1000, sc‐47724), and H2A (1:1000, sc‐517336) were purchased from Santa Cruz (Santa Cruz, CA, USA). The antibody against P‐gp (1:1000, 12683) was purchased from the Cell Signalling Technology (Boston, MA, USA). The antibody against Sp3 (1:1500, PA5‐59159) was purchased from the ThermoFisher. After washed with PBS three times, membrane was incubated with appropriate secondary antibody (Abcam, Cambridge, UK) for 1 hour at 37°C. The protein was measured by a chemiluminescence reagent (Pierce, Rockford, IL, USA) and normalized to the expression of GAPDH. To measure the sub‐cellular distribution of proteins, the nucleus and cytosol was separated by use of the NE‐PER Nuclear and Cytoplasmic Extraction Kit (Thermo Fisher Scientific, Inc., Pierce, Waltham, MA, USA).

### 2.6 Cell transfection

The negative control, siRNAs for ERRα and SP3, and miR‐9 inhibitor and mimic were purchased from Guangzhou Ribo BioCoLTD (Guangzhou, China). Cells plated in six‐well plates (6 × 10^5^ cells/well) were transfected with siRNAs (working concentration 50 nmol/L) diluted in serum‐free medium by use of lipofectamine 2000 (Invitrogen, Long Island, NY, USA) according to the manufacturer's protocol. After transfection for 6 hours, the medium was replaced by full medium containing 10% FBS.

### 2.7 Dox efflux assay

Effects of ERRα on the Dox efflux were tested by use of flow cytometry according to the previous study.^27^ Cells were treated with 5 μmol/L Dox for 1 hour in darkness at 37°C. After treatment, cells were washed and collected for analysis by use of a flow cytometer (BD Biosciences, San Jose, CA, USA) using an argon laser of 15 mW at 488 nm.

### 2.8 Promoter activity assay

The promoter of ABCB1 (−1000 to −1 bp) was cloned to the luciferase promoter to generate pTL‐MDR1. Cells were transfected with pTL‐MDR1 and pBABE‐puro using Lipofectamine 2000 reagent (Invitrogen) and further treated with or without XCT‐790 for the indicated time periods, the luciferase activity was normalized to total proteins determined by use of BCA assay.

### 2.9 Immunoprecipitation assay

The binding between ERRα and SP3 was measured by immunoprecipitation assay in OS cells. Cell lysis was incubated with IgG or ERRα antibody over night at 4°C. After further incubated with protein A agarose beads for 4 hours, the immunoprecipitate was washed for three times and subjected to western blot analysis. The antigen‐antibody complexes were visualized by chemiluminescence.

### 2.10 Statistical analysis

All experiments were repeated at least three times. The data were analyzed using SPSS 18.0 software package (SPSS, Chicago, IL, USA). Student's *t* test was used to analyze the difference between two groups. The *P* value <0.05 were considered statistically significant.

## 3 RESULTS

### 3.1 The establishment of OS/Dox and OS/CDDP cells

**Figure 1:**
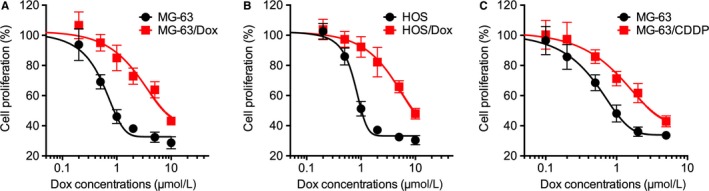
The establish of OS/Dox and OS/CDDP cells. MG‐63/Dox (A) or HOS/Dox (B) cells and their parental cells were treated with increasing concentrations of Dox for 48 h; (C) MG‐63/CDDP and MG‐63 cells were treated with increasing concentrations of CDDP for 48 h. Data are presented as means ± SD of three independent experiments

Firstly, the sensitivity of MG‐63 and HOS parental and Dox resistant cells were evaluated by use of CCK‐8 kits. Our data showed that the established Dox resistant cells were much more resistant to Dox treatment as compared to their corresponding parental cells. The IC_50_ values of Dox for MG‐63/Dox and MG‐63 were 7.56 and 0.81 μmol/L, respectively (Figure 1A). The IC_50_ values of Dox for HOS/Dox and HOS were 9.25 and 0.96 μmol/L, respectively (Figure 1A). Similarly, the established CDDP resistant MG‐63 cells (MG‐63/CDDP, IC_50_ 7.93 μmol/L) were much more resistant to CDDP treatment as compared to the parental cells (IC_50_ 0.91 μmol/L).

### 3.2 ERRα was up‐regulated in chemoresistant OS cells

**Figure 2:**
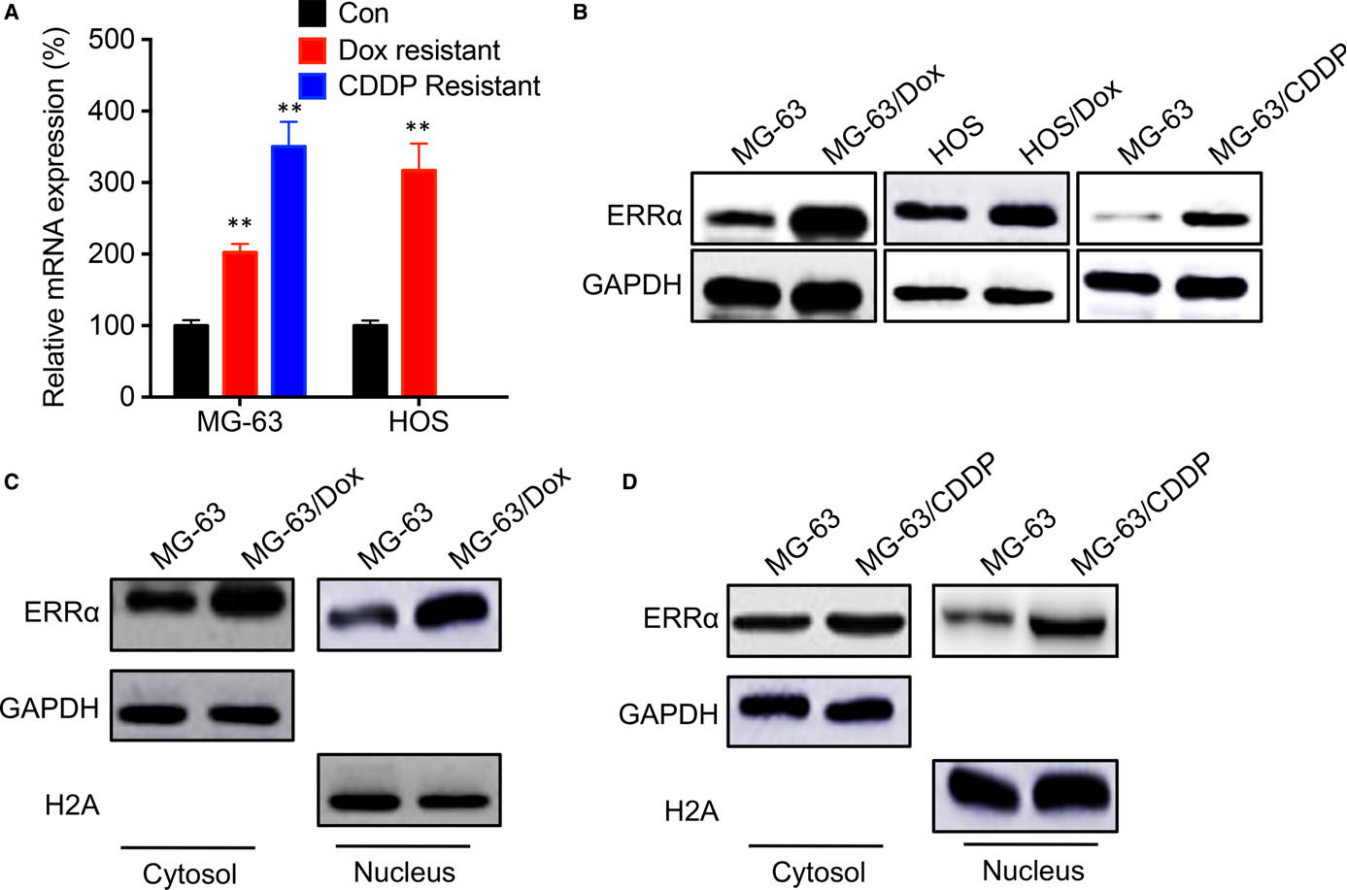
Oestrogen‐related receptors alpha (ERRα) was up‐regulated in OS chemoresistant cells. The mRNA (A) or protein (B) expression of ERRα in OS chemoresistant or parental cells were checked by qRT‐PCR or western blot analysis, respectively; The subcellular localization of ERRα in MG‐63/Dox (C), MG‐63/CDDP (D) or parental cells was checked by western blot analysis. Data are presented as means ± SD of three independent experiments. **P <* *0.01 compared with control

Our previous study showed that ERRα participates TGF‐β induced EMT of OS cells.^25^ We then checked the expression of ERRα in OS resistant and their parental cells. qRT‐PCR showed that the mRNA expression of ERRα was significantly increased in MG‐63/Dox, MG‐63/CDDP and HOS/Dox cells as compared with their control cells (Figure 2A). Consistently, Western blot analysis confirmed that the protein expression of ERRα was increased in MG‐63/Dox, MG‐63/CDDP and HOS/Dox cells as compared with their control cells (Figure 2B). Subcellular fraction analysis showed that the nucleus accumulation of ERRα was increased in both MG‐63/Dox (Figure 2C) and MG‐63/CDDP (Figure 2D) cells as compared with MG‐63 cells.

### 3.3 ERRα was involved in the chemoresistance of OS cells

**Figure 3:**
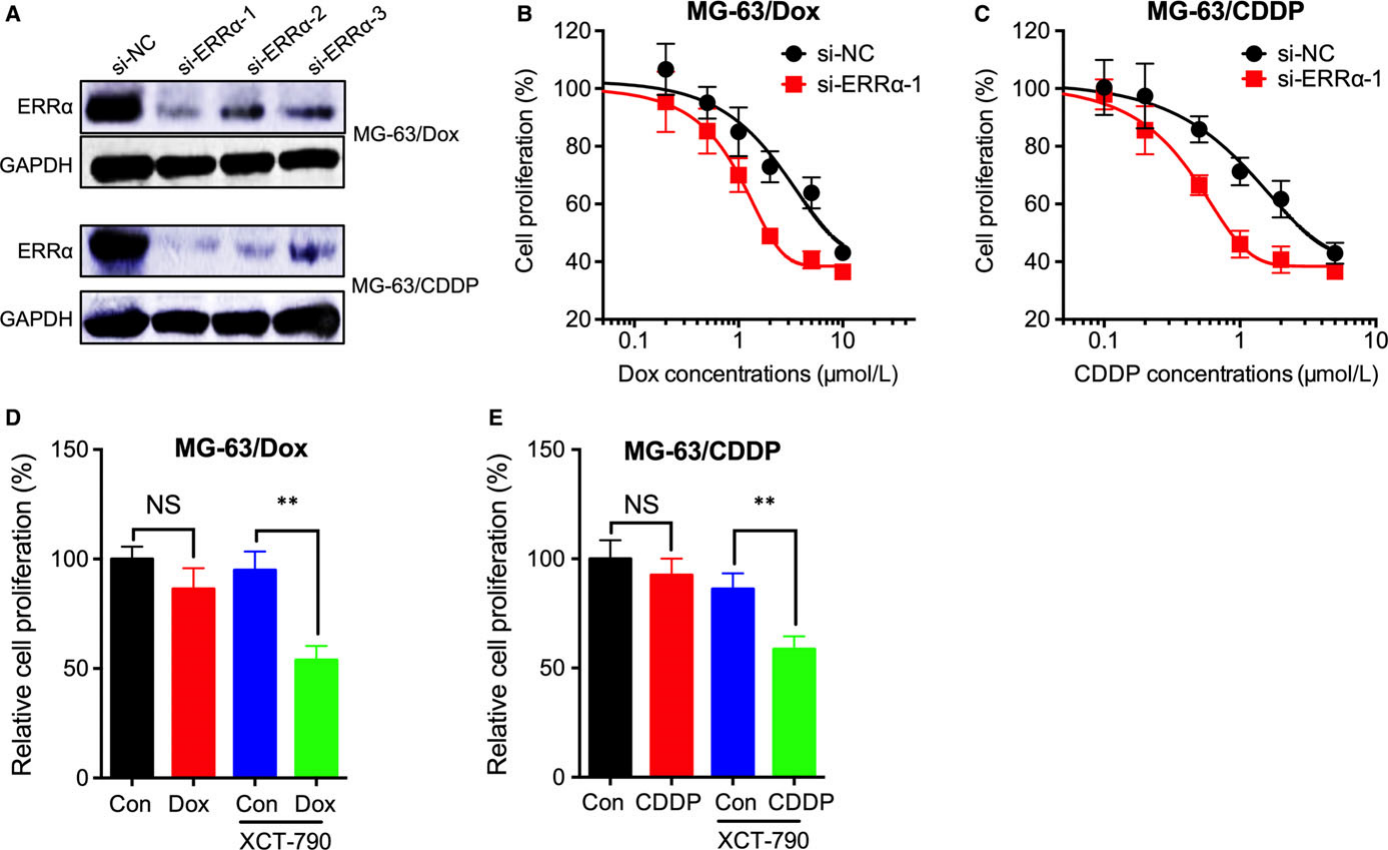
Oestrogen‐related receptors alpha (ERRα) was involved in the chemoresistance of OS cells. MG‐63/Dox or MG‐63/CDDP cells were transfected with siRNA negative control (si‐NC) or siRNAs for ERRα for 24 h, the expression of ERRα was checked by western blot analysis. Si‐ERRα‐1 was used for the next studies; MG‐63/Dox (B) or MG‐63/CDDP (C) cells were transfected with siRNA negative control (si‐NC) or siRNAs for ERRα for 12 h and then further treated with increasing concentrations of Dox or CDDP for 48 h; (D) MG‐63/Dox cells were treated with 1 μmol/L of Dox together with or without 1 μmol/L XCT‐790 for 48 h; (D) MG‐63/CDDP cells were treated with 0.5 μmol/L of CDDP together with or without 1 μmol/L XCT‐790 for 48 h. Data are presented as means ± SD of three independent experiments. **P <* *0.01 compared with control

In order to investigate whether ERRα was involved in the chemoresistance of OS cells, MG‐63/Dox or MG‐63/CDDP cells were transfected with si‐ERRα (Figure 3A). Our results suggested that si‐ERRα can significantly increase the sensitivity of MG‐63/Dox cells to Dox treatment (Figure 3B). Similarly, si‐ERRα can significantly increase the sensitivity of MG‐63/CDDP to CDDP treatment (Figure 3C). We further treated MG‐63/Dox cells with XCT‐790, the inverse agonist of ERRα.^24^ XCT‐790 can also increase the sensitivity of MG‐63/Dox (Figure 3D) and MG‐63/CDDP (Figure 3E) to chemotherapy treatment.

### 3.4 ERRα regulated the expression of P‐gp in OS chemoresistant cells

**Figure 4:**
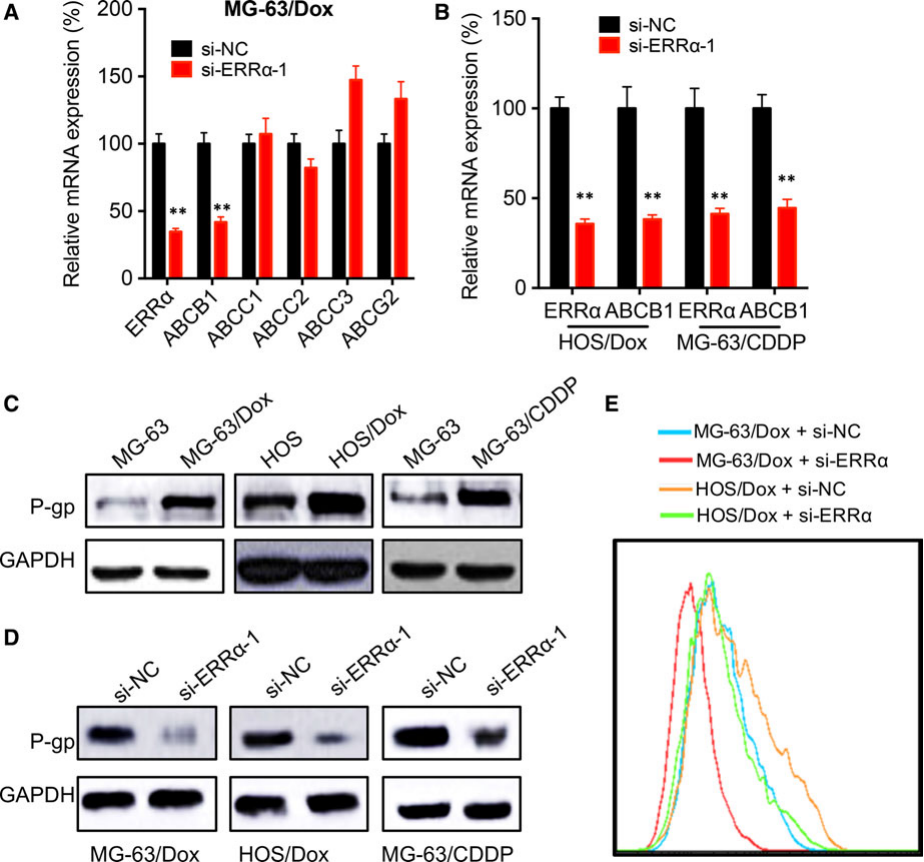
Oestrogen‐related receptors alpha (ERRα) regulated the expression of P‐gp in OS chemoresistant cells. MG‐63/Dox cells were transfected with si‐NC or siRNAs for ERRα for 24 h, the expression of ABC membrane transporters was checked by qRT‐PCR. (B) HOS/Dox or MG‐63/CDDP were transfected with si‐NC or siRNAs for ERRα for 24 h, the expression of ABCB1 was checked by qRT‐PCR. (C) The expression of P‐gp in OS chemoresistant and parental cells were checked by western blot analysis. (D) Cells were transfected with si‐NC or siRNAs for ERRα for 24 h, the expression of P‐gp was checked; (E) MG‐63/Dox and HOS/Dox cells were transfected with si‐NC or si‐ERRα for 24 h and then further exposed treated with 1 μmol/L Dox for 8 h, the cell retention of Dox was detected by flow cytometry. Data are presented as means ± SD of three independent experiments. **P <* *0.01 compared with control

We further checked the effects of ERRα on the expression of various ABC membrane transporters including ABCB1, ABCC1, ABCC2, ABCC3 and ABCG2 in MG‐63/Dox cells by transfection of si‐ERRα. Our data showed that si‐ERRα can significantly inhibit the mRNA expression of ABCB1 in MG‐63/Dox cells (Figure 4A). Further, si‐ERRα also significantly decreased the mRNA expression of ABCB1 in HOS/Dox and MG‐63/CDDP cells (Figure 4B). Western blot confirmed that the expression of P‐gp was increased in MG‐63/Dox, HOS/Dox and MG‐63/CDDP cells as compared with their corresponding controls (Figure 4C). Furthermore, si‐ERRα can decrease the expression of P‐gp in MG‐63/Dox, HOS/Dox and MG‐63/CDDP cells (Figure 4D). In addition, si‐ERRα can increase the Dox retention in MG‐63/Dox cells (Figure 4E), which further confirmed the roles of ERRα on expression of P‐gp.

### 3.5 ERRα regulated the transcription and mRNA stability of ABCB1

**Figure 5:**
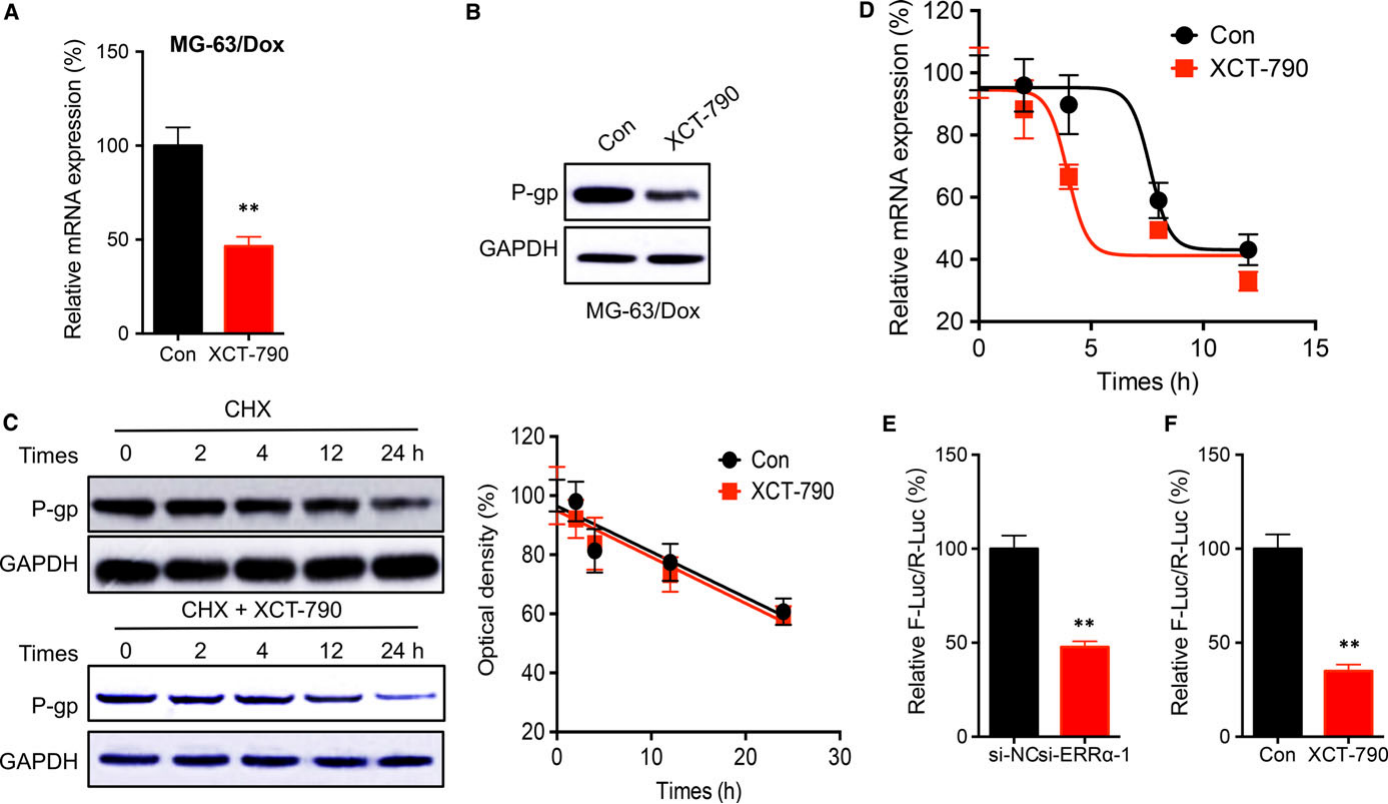
Oestrogen‐related receptors alpha (ERRα) regulated the transcription and mRNA stability of ABCB1. MG‐63/Dox cells were treated with or without 1 μmol/L XCT‐790 for 24 h, the mRNA (A) and protein (B) levels of P‐gp was measured, respectively; (C) MG‐63/Dox cells were treated with 10 μg/mL CHX together with or without 1 μmol/L XCT‐790 for the indicated time periods, the expression of P‐gp was measured by western blot analysis; (D) MG‐63/Dox cells were treated with 5 μg/mL Act‐D together with or without 1 μmol/L XCT‐790 for the indicated time periods, the expression of ABCB1 was measured; MG‐63/Dox cells were treated with si‐ERRα (E) or XCT‐790 (1 μmol/L) (F) for 24 h, the relative promoter activity of ABCB1 was analyzed by a dual‐luciferase assay kit. Data are presented as means ± SD of three independent experiments. **P <* *0.01 compared with control

Mechanisms responsible for ERRα regulated expression of ABCB1 were further investigated. Our study revealed that XCT‐790 can decrease the expression of mRNA (Figure 5A) and protein (Figure 5B) levels of P‐gp in MG‐63/Dox cells. By treating cells with translation inhibitor CHX, our data showed that XCT‐790 had no effect on the protein stability of P‐gp in MG‐63/Dox cells (Figure 5C). By treating cells with transcription inhibitor Act‐D, our data showed that XCT‐790 can significantly decrease the half‐life of ABCB1 mRNA in MG‐63/Dox cells (Figure 5D). Further, dual luciferase assay showed that both XCT‐790 (Figure 5E) and si‐ERRα (Figure 5F) can decrease the promoter activity of ABCB1. These results suggested that ERRα regulated the transcription and mRNA stability of ABCB1.

### 3.6 ERRα bound to SP3 to increase the transcription of ABCB1

**Figure 6:**
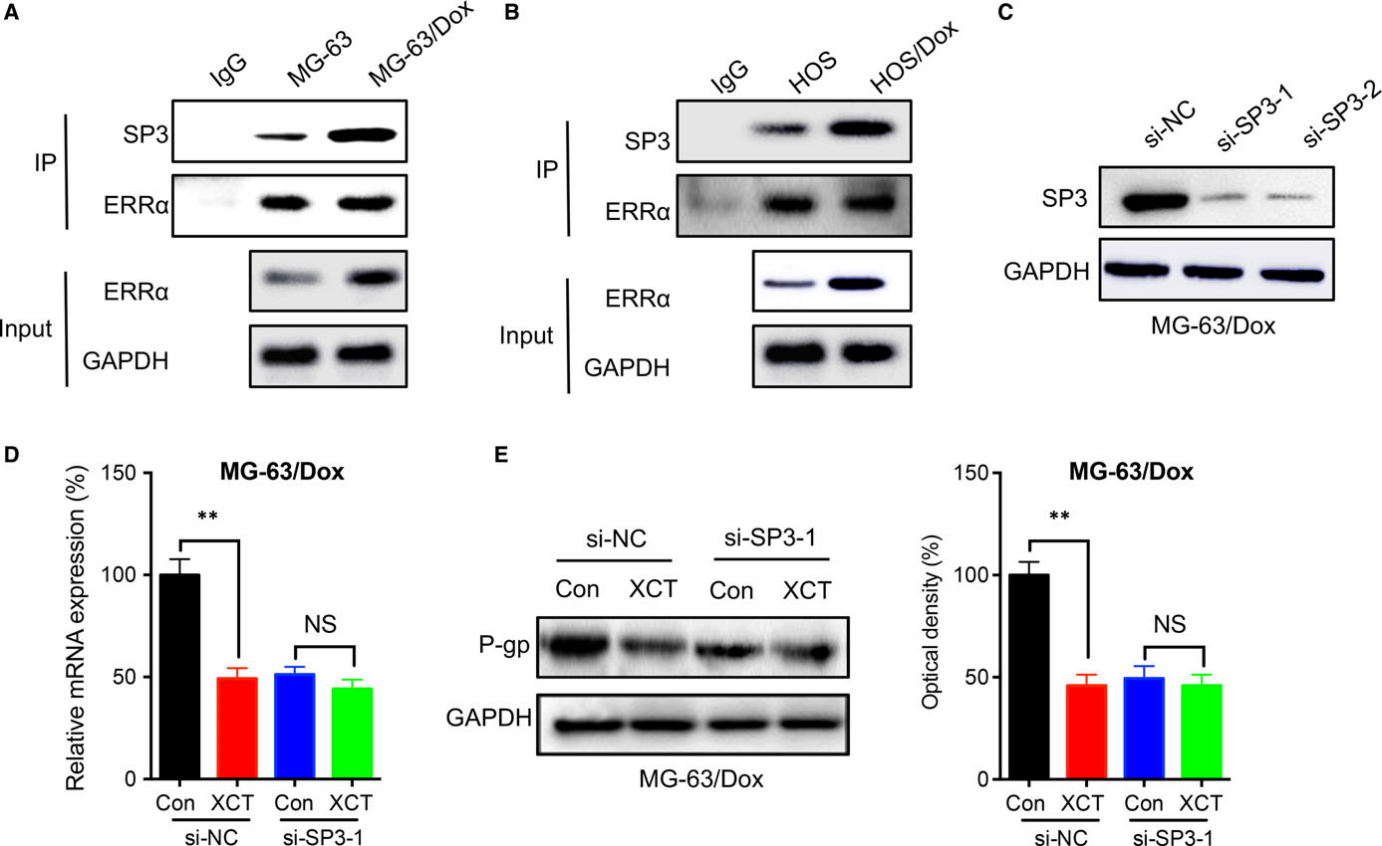
Oestrogen‐related receptors alpha (ERRα) bound to SP3 to increase the transcription of ABCB1. The ERRα was immunoprecipitated by use of its specific antibody in MG‐63/Dox (A) or HOS/Dox (B) and their parental cells, respectively. The bound SP3 was measured by western blot analysis, IgG was used as the negative control; (C) MG‐63/Dox cells were transfected with si‐NC or si‐SP3 for 24 h; MG‐63/Dox cells were transfected with si‐NC or si‐SP3 for 12 h and then further treated with or without 1 μmol/L XCT‐790 for 24 h, the mRNA (D) or protein (E) expression of P‐gp was measured and quantitatively analyzed. Data are presented as means ± SD of three independent experiments. **P <* *0.01 compared with control

It has been suggested that SP3 can regulate the transcription of ABCB1 in various human tissues.^28^ We then investigated SP3 was involved in ERRα regulated transcription of ABCB1. The results showed that ERRα can directly bind with SP3 in MG‐63 cells, further, the binding between ERRα and SP3 was increased in MG‐63/Dox cells (Figure 6A). Similarly, the binding between ERRα and SP3 was increased in HOS/Dox cells as compared with that in HOS cells (Figure 6B). We then knocked down the expression of SP3 in MG‐63/Dox cells by use of its specific siRNA (Figure 6C). Our data showed that in cells transfected with si‐SP3, the XCT‐790 suppressed transcription of ABCB1 was abolished (Figure 6D). Furthermore, Western blot analysis confirmed that si‐SP3 can attenuate XCT‐790 suppressed expression of P‐gp (Figure 6E). These results suggested that ERRα can bind to SP3 to increase the transcription of ABCB1.

### 3.7 miR‐9 was involved in ERRα regulated mRNA stability of ABCB1

**Figure 7:**
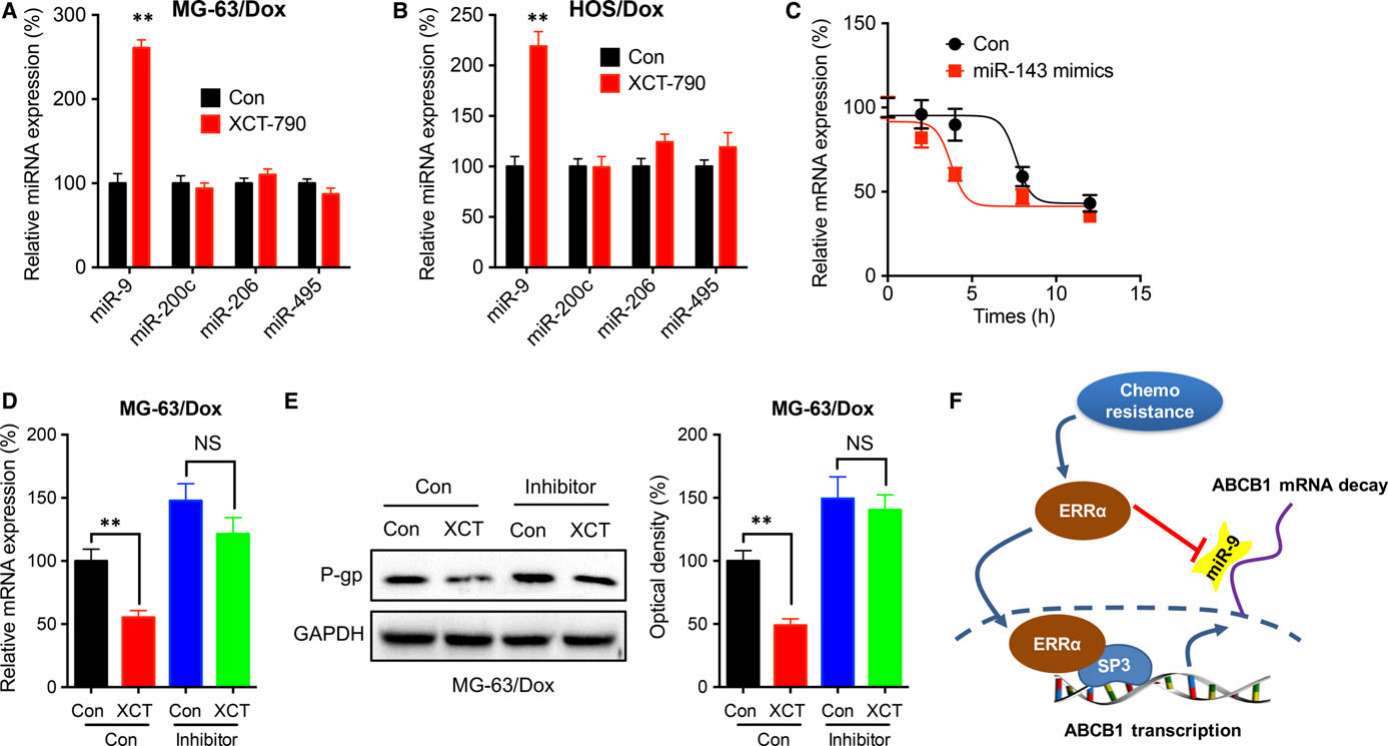
miR‐9 was involved in oestrogen‐related receptors alpha (ERRα) regulated mRNA stability of ABCB1. MG‐63/Dox (A) or HOS/Dox (B) were treated with or without 1 μmol/L XCT‐790 for 24 h, the expression of miRNAs was measured; (C) MG‐63/Dox cells were transfected with RNA scramble control or miR‐9 mimics for 12 h and then further treated with Act‐D for the indicated times, the mRNA of ABCB1 was measured; MG‐63/Dox cells were transfected with si‐NC or miR‐9 inhibitor for 12 h and then further treated with or without 1 μmol/L XCT‐790 for 24 h, the mRNA (D) or protein (E) expression of P‐gp was measured and quantitatively analyzed. (F) The schematic of ERRα regulated ABCB1 expression via binding with SP3 and suppressing miR‐9 expression in OS cells. Data are presented as means ± SD of three independent experiments. **P <* *0.01 compared with control

Since ERRα can regulate the mRNA stability of ABCB1, we then investigated whether miRNAs were involved in this process. The expression of miR‐9,^26^ miR‐200c,^29^ miR‐206,^30^ and miR‐495^31^ was investigated in cells treated XCT‐790 due to they can directly bind to 3′UTR of ABCB1 to regulate its stability. Our data showed that XCT‐790 can increase the expression of miR‐9, while not others, in both MG‐63/Dox (Figure 7A) and HOS/Dox (Figure 7B) cells. The mimics of miR‐143 can decrease the half‐life of ABCB1 mRNA in MG‐63/Dox cells (Figure 7C). Further, the inhibitor of miR‐9 can abolish XCT‐790 suppressed expression of ABCB1 in MG‐63/Dox (Figure 7D). Western blot confirmed that inhibitor of miR‐9 attenuated XCT‐790 suppressed expression of P‐gp in MG‐63/Dox (Figure 7E). These data suggested that miR‐9 was involved in ERRα regulated mRNA stability of ABCB1.

## 4 DISCUSSION

Although ERRα has been reported to promote the progression of cancers, its roles in chemotherapy resistance are rarely investigated. Our study showed that the expression of ERRα was up‐regulated in OS chemoresistant cells such as MG‐63/Dox, HOS/Dox and MG‐63/CDDP. Targeted inhibition of ERRα can restore the sensitivity of OS resistant cells to chemotherapy. This might be due to that inhibition of ERRα can down‐regulate the expression and function of P‐gp via decreasing its transcription and mRNA stability. In OS resistant cells, ERRα can bind to SP3 to regulate its transcription. Furthermore, ERRα can regulate miR‐9 induced decay of ABCB1 mRNA. Taken together, our study revealed that ERRα can regulate the chemoresistance of OS cells via regulating the transcription and mRNA stability of ABCB1 (Figure 7F).

ERRα has been reported to regulate the proliferation, migration and epithelial to mesenchymal transition of cancer cells.^32^, ^33^ Recently, studies also suggested that ERRα mediates metabolic adaptations driving drug resistance in breast cancer.^23^, ^34^ Our study revealed that the expression of ERRα was up‐regulated in OS resistant cells as compared to their parental cells. This might be due to the activation of its transcription in OS resistant cells since mRNA of ERRα was increased. It has been suggested that the biarylpyrazole compound AM251 can trigger the proteolytic degradation of ERRα.^35^ Whether post‐translation modification is involved in the up‐regulation of ERRα in chemoresistant OS cells needs further studies. Further, targeted inhibition of ERRα via its inverse agonist or siRNAs can restore the chemotherapy sensitivity. This was consistent with recent study that ERRα can confer methotrexate resistance of OS cells.^21^ It has been reported that ERRα is a transcriptional activator during bond development^36^ and can regulate the expression of osteopontin in OS cells.^37^ Our previous study also suggested that ERRα is involved in TGF‐β induced EMT of OS cells.^25^ All these data indicated that targeted inhibition of ERRα can suppress the malignancy of OS and might be a potential therapy approach for OS patients.

Our data showed that ERRα can regulate the expression of P‐gp via suppression its transcription and regulating its mRNA stability. Over expression of P‐gp is one of the major causes of drug resistance for various cancer cells including OS.^38^ Inhibition the expression of ABCB1 (P‐gp) can overcome the drug resistance of OS cells.^39^ We found that ERRα can bind with SP3 to regulate the transcription of ABCB1 in OS resistant cells. It has been reported that SP3 may recruit TFIID to the ABCB1 promoter by binding to the second activation domain of Sp3.^28^ In our study, XCT‐790 suppressed transcription of ABCB1 was abolished in cells transfected with si‐SP3. In addition, miR‐491‐3p, which can down‐regulate the expression of ABCB1 and its transcription factor Sp3 by directly targeting their 3′‐UTR, attenuated multidrug resistance of hepatocellular carcinoma.^40^ Furthermore, we found that XCT‐790 can increase the expression of miR‐9 and then trigger the decay of ABCB1 mRNA. Various miRNAs such as miR‐9,^26^ miR‐200c,^29^ miR‐206,^30^ and miR‐495^31^ can regulate the expression of ABCB1 in cancer cells. It has been confirmed by luciferase activity analysis that miR‐9 can direct bind to the 3′UTR of ABCB1 and then trigger its decay in cancer cells.^26^ As a transcription factor, ERRα might occupy the conserved ERRE in the promoter of miRNAs and then regulate their expression.^41^ The mechanisms that XCT‐790 induced up‐regulation of miR‐9 should be further investigated in the future.

In summary, our study suggested that ERRα mediated the chemoresistance of OS cells via regulating the transcription and mRNA stability of ABCB1. Although further in vivo and clinical studies are needed to confirm the clinical significances, our data, together with published data, suggested that targeted inhibition of ERRα can suppress the malignancy of OS and might be a potential therapy target of OS.

## DECLARATIONS

### Ethics approval and consent to participate

No human or animal study in this study.

### Consent for publication

All authors give the consent for the publish of this study.

### Availability of data and material

All data and material are available.

### Disclosure of potential conflicts of interest

The authors declare no conflict of interest.

## AUTHOR’S CONTRIBUTIONS

Data collecting: YC, KZ, YL; writing: YC, KZ, GZ, QH; data analysis: YL, RG, KZ, GZ, QH; design: YC, KZ, GZ, QH.
